# Masticatory Muscle Pain and Associated Complaints—An Analysis of the Frequency and Coexistence of Symptoms Before and During the COVID-19 Pandemic

**DOI:** 10.3390/jcm14134473

**Published:** 2025-06-24

**Authors:** Zofia Maciejewska-Szaniec, Barbara Maciejewska, Małgorzata Gałczyńska-Rusin, Weronika Jakubowska, Natalie Górna, Izabela Maćkowiak, Tomasz Gedrange, Marta Kaczmarek-Ryś, Agata Czajka-Jakubowska

**Affiliations:** 1 Department of Orthodontics and Temporomandibular Disorders, University of Medical Sciences, Bukowska Street 70, 60-812 Poznan, Poland; zofiamaciejewska@wp.pl (Z.M.-S.); mgalczynskarusin@ump.edu.pl (M.G.-R.); natalie.nejsi@gmail.com (N.G.); izabela.mackowiak99@gmail.com (I.M.); agatacj@gmail.com (A.C.-J.); 2 Department and Clinic of Phoniatrics and Audiology, University of Medical Sciences, Przybyszewskiego Street 49, 60-356 Poznan, Poland; barbaramaciejewska@ump.edu.pl; 3 Department of Risk Group Dentistry, University of Medical Sciences, Bukowska Street 70, 60-812 Poznan, Poland; jakubowskaweronika95@gmail.com; 4 Department and Institute of Oral Surgery, Wroclaw Medical University, Krakowska Street 26, 50-425 Wroclaw, Poland; 5 Institute of Human Genetics, Polish Academy of Sciences, Strzeszyńska Street 32, 60-479 Poznan, Poland; m.kaczmarekrys@gmail.com

**Keywords:** masticatory muscles, pain, headache, tinnitus, COVID-19

## Abstract

**Background/Objectives**: Chronic stress has an undeniable effect in generating emotional disorders and physiological changes. It results in excessive muscle tension throughout the body, also in the masticatory system. A situation of chronic stress was the COVID-19 pandemic. The aim of this paper was to assess the prevalence of specific masticatory pain symptoms, their severity, and the co-occurrence of associated symptoms (otological symptoms and headaches) in patients diagnosed before and during the COVID-19 pandemic. **Methods**: A total of 202 patients were divided into two groups: Group A (mean age of 36.46; F = 64; and M = 37) and B (mean age of 26.04; F = 70; and M = 31) included patients who presented for the study before and after the COVID-19 pandemic, respectively. The Oral Behaviours Checklist (OBC) questionnaire was used: patients with result ≥2 scores in the OBC were evaluated by DC/TMD. To evaluate the intensity of pain in masticatory structures, the elements of the RDC-TMD questionnaire were used. Otologic symptoms and headaches were assessed as coexisted complaints. **Results**: A significant increase in pain occurrence was observed in Group B mainly for masseter muscles (*p* < 0.0001), temporalis (*p* = 0.0044), and medial pterygoid muscles (*p* = 0.0153). A significantly more frequent reporting of pain/tenderness was observed among men in most of the evaluated muscles. For the lateral pterygoid muscles, changes in palpation pain did not reach statistical significance. There was a statistically significant difference in the intensity of pain in the temporomandibular joint area between both the entire groups A and B (*p* = 0.000152), as well as between women in Group A and B (*p* = 0.006453) and men in the study groups (*p* = 0.007990). An increase in the incidence of headaches was observed among men in Group B (Group A with 40.6% vs. Group B with 67.3%). The most commonly reported otological symptom in both groups was ear pain and/or discomfort in the preauricular region, with the frequency of otological symptoms being higher in Group B. **Conclusions**: (1) The COVID-19 pandemic affected the incidence and severity of masticatory muscle pain and associated complaints. (2) A decrease in the age of patients reporting complaints of masticatory mm pain was observed during the COVID-19 pandemic. (3) An increase in the frequency of headaches was observed in the male group during the pan-demic, while in women there was an increase in palpation tenderness of masticatory muscles.

## 1. Introduction

The introduction of the COVID-19 pandemic state by the World Health Organization (WHO) on 11 March 2020 triggered mass fear on an unprecedented scale throughout the world due to the health, economic, and social consequences of already existing infections and of potentially new infections. The sense of threat for one’s own and loved ones’ health and life, isolation, restriction or even complete lack of contact with family and friends, and feelings of helplessness and growing frustration or fear of losing one’s job became the cause of chronic stress regardless of age, gender, religion, or level of education [1,2]. The COVID-19 pandemic has adversely affected the psycho-emotional condition of people around the world [2]. Long-term stress triggers the body’s defensive reactions, generating various degrees of pathological emotional tension [3,4]. Studies have shown higher muscle activity in the swelling of neck and head muscles, contributing to the generation of tension headaches and migraines [5,6]. Increased masticatory muscle tension may be one of the factors predisposing people to temporomandibular disorders (TMD) and bruxism—clenching or grinding of the teeth and/or by bracing or thrusting of the mandible [7]. The association between the co-occurrence of bruxism and temporomandibular disorders is highlighted in a meta-analysis [8,9]. The increased resting and functional activity of the masticatory muscles (especially the masseter and temporalis muscles) in the COVID-19 pandemic may have been influenced by the use of protective masks recommended by the WHO, although scientific reports on this topic are inconclusive [10]. Remote work may have had a similar effect. Excessive use of computers, tablets, and smartphones promoted prolonged abnormal posture, mainly of the head and neck (forward extension). Increased masseter muscle tension has been reported in excessive users of said electronic devices [11]. Chronic stress on the muscles of mastication, especially the masseter muscle, results not only in subjective symptoms (pain, tenderness, and hypertrophy), but also in objective changes at the tissue level [12]. Within the masseter muscle itself, changes have been observed in the average area of the muscle and its thickness, which can be assessed by imaging studies (MRI and ultrasonography) [13,14,15]. Histological and histochemical results also show changes at the cellular level under the influence of changes in tension and load due to the distribution of muscle fibre types. It should be noted that the fibre composition of the human jaw muscles differs from that of the limb muscle fibres. Jaw muscle fibres are hybrid, i.e., they contain more than one type of MyHC, myosin heavy chain isoform, which influences the high gradation capacity of muscle tensions [16,17,18]. Jaw muscles notably present more hybrid fibres, containing more than one MyHC isoform, than limb or trunk muscles. These hybrid fibres have contractile properties that differ from pure fibre, and present intermediate characteristics from each of the MyHC isoforms they express. Thus, hybrid fibre expressing both MyHC-I and IIA, for instance, will be faster than pure MyHC-I fibre but slower than pure MyHC-IIA fibre [19]. The literature has shown that both excessive stress on the masticatory muscles and a reduction in their function lead to changes in muscle fibre size and quantitative changes in their subtypes [17,20,21,22]. In addition, changes in adjacent/neighbouring structures are often observed in the course of chronic masticatory muscle stress. Between the structures of the masticatory organ and the middle ear, there are correlations at the embryological, anatomical, and physiological levels. These factors are thought to potentially generate the co-occurrence of masticatory organ disorders and otologic complaints [23].

Accordingly, the authors hypothesized that masticatory muscle tension and pain would be more frequent and of greater severity in patients tested during the SARS-CoV-2 pandemic compared to patients tested before the COVID-19 outbreak.

## 2. Materials and Methods

### 2.1. Participants

The study group consisted of 202 patients (134 females and 68 males, aged 18–40 years) who visited the Department of Orthodontics and Temporomandibular Disorders at the Poznan University of Medical Sciences for the diagnosis and treatment of masticatory pain. The study was conducted by two doctors with relevant qualifications (specialization in dental prosthodontics) and more than 10 years of clinical experience with patients suffering from functional disorders of the masticatory organ.

Patients were divided into 2 groups based on the date of the visit. Group A (mean age of 36.46; F = 64; and M = 37) included patients who presented for the study before the COVID-19 pandemic was announced (i.e., during 01/2018–01/2019); Group B (mean age of 26.04; F = 70; and M = 31) consisted of patients who presented during 07/2020–07/2021, i.e., during the COVID-19 pandemic in Poland, but a minimum 3 months after its announcement.

The participants were adults, with full dental arches, preserved support zones and the acceptable absence of third molars, with interdental contacts in Angle I class. Patients who were qualified for the study had not previously attempted pharmacological or rehabilitative treatment for their reported masticatory complaints.

The exclusion criteria included the following: age over 40 years, secondary bruxism caused by neurological disorders and/or neuropathic pain (epilepsy, vagus nerve neuropathy, myasthenia gravis, and Parkinson’s disease), use of drugs and substances that can significantly affect the functioning of the nervous and muscular systems (e.g., myorelaxants and psychostimulants), mental disorders, cancer, pacemaker, pregnancy, lactation, intellectual disability, and history of facial trauma and surgery.

The patients were healthy, and none of them showed obvious symptoms related to SARS-CoV-2 at the time of the study. In accordance with the procedure at the time, patients were asked to present a negative screening test result before the study at the clinic.

Prior to entering the study, all participants were provided with detailed information on the study’s objectives and procedures and gave informed written consent to participate in the study. All participants were informed of the study’s purpose and procedures and gave informed written consent to participate in the study. The study was approved by the Bioethics Committee of the Poznan University of Medical Sciences. (Resolution No. 741/16).

### 2.2. Methods

The Oral Behaviours Checklist (OBC) questionnaire [24] was used as a screening tool to assess the frequency of specific oral behaviours (parafunctions). Responses were assessed using a 5-point Likert scale, where “never” was assigned a score of 0 and “always” a score of 5. Participants who scored ≥2 were qualified for the study.

### 2.3. Dental Examination

The examination included an evaluation of hyperfunction, tenderness, or pain in the muscles of the masticatory organ. All patients were evaluated according to the Diagnostic Criteria for Temporomandibular Disorders (DC/TMD) questionnaire [25]. The Research Diagnostic Criteria for Temporomandibular Disorders (RDC-TMD) questionnaire items were used to assess the intensity of pain in the masticatory structures [26]. The palpable pain of the muscles of the masticatory organ, such as the masseter, temporalis muscle, medial pterygoid muscle, and lateral pterygoid muscle, was assessed. The average intensity of muscle pain in each group was assessed by evaluating the tenderness of both muscles (right and left combined). In addition, the examination included an assessment of pain in the temporomandibular joint area and temporomandibular joint noises during opening and closing.

### 2.4. Evaluation of Other Symptoms

Participants completed a questionnaire to detect associated symptoms such as tinnitus, pressure in the ear, ear pain, and hearing loss and dizziness (otologic symptoms) in the past 30 days.

A four-point scale was used to assess patients’ headache in the past 30 days, with gradations identical to those in RDC/TMD, where 0 = no pain/pressure only, 1 = mild pain, 2 = moderate pain, and 3 = severe pain. Patients with a history of headaches underwent further neurological diagnosis. All patients with such symptoms underwent an otoscopy, acumetric assessment, and fork test for hearing screening by an audiologist working with the dental team.

### 2.5. Statistical Analysis

Patients qualified for the study had not previously attempted any pharmacological or rehabilitative treatment so far. All subjects were informed about the purpose and conduct of the study before the start of the study. Written informed consent to participate was given.

Statistical analyses of all the collected data were carried out using Statistica PL, Cloud Software Group, Inc., Fort Lauderdale, FL, USA, (2023). Data Science Workbench, version 14. http://tibco.com (accessed on 14 May 2025). To compare the categorical data, we used the chi-square test for independence or alternatively the Fisher exact test if 20% of expected values were below 5 (Cochrane condition). Tests were considered as significant at *p* < 0.05. If a test involved multiple comparisons, significance level was counted separately for each using the Bonferroni correction.

## 3. Results

### 3.1. Masticatory System

Differences in pain intensity were noted in the muscles assessed, and the results are shown in Table 1 and Table 2. A significant increase in pain intensity was observed in patients during COVID-19 (Group B), primarily in the masseter and temporalis muscles. The former were more likely to become tender/painful (0 vs. 1; 0 vs. 2; and 0 vs. 3), while the latter were significantly more likely to reach a maximum pain intensity (1 vs. 3 and 2 vs. 3). Less pronounced changes involved the medial pterygoid muscles, while, for the lateral pterygoid muscles, changes in palpation pain did not reach statistical significance. Considering gender differences, a significantly more frequent reporting of pain/tenderness was observed among men, in muscles that had not previously shown complaints (masseter, temporalis, and medial pterygoid muscles) (0 vs. 1 + 2 + 3). Changes in pain intensity were mainly observed for the temporalis and medial pterygoid muscles, with little change for the masseter and lateral pterygoid muscles.

Women in Group A already showed muscle tenderness in the masseter and temporalis muscles, and this tenderness increased during the pandemic period. Soreness appeared significantly more frequently in the medial pterygoid mm (0 vs. 1.0 vs. 2.0 vs. 3), but there were no differences in intensity (1 vs. 2; 1 vs. 3; and 2 vs. 3). There were no statistically significant differences in lateral pterygoid muscles.

At the first visit before the pandemic, women presented with masseter and temporal mm pain but of lesser intensity than at the time of the pandemic (escalation of pain predominated). In contrast, men before the pandemic did not present with pain sensations, while during COVID-19 they reported and appeared with palpable masseter and temporal mm pain. There was statistically noticeable tenderness of the medial pterygoid muscles in both groups, with no difference for the lateral pterygoid muscles.

There was a statistically significant difference in the intensity of pain in the temporomandibular joint area between Group A and B (*p* = 0.0002). The differences were observed between women in Group A and B (*p* = 0.0065) and men in the study groups (*p* = 0.007990) (based on data from Table 3). However, there were no statistical differences within groups between genders (Group A with *p* = 0.4661 and Group B with *p* = 0.8305).

Analysis of the incidence of acoustic symptoms at the temporomandibular joint during mandibular inversion and adduction movements showed no significant statistical differences, either between whole groups or by gender within groups and between mini-groups (Table 4 and Table 5).

### 3.2. Otologic Symptoms

The incidence of otologic symptoms increased in Group B, in both women and men, but the values did not reach statistical significance. The most commonly reported otologic symptom in both groups was ear pain and/or discomfort in the preauricular area (Table 6).

### 3.3. Headache

Headache severity was generally mild (1) to moderate (2), with no statistically significant differences between groups (*p* < 0.05) (Table 7). However, an increase in the incidence of headaches was observed among men in Group B (Group A with 40.59% vs. Group B with 67.33%) (Table 8).

## 4. Discussion

The results of Saccomanno’s work indicate that the anxiety, uncertainty, and isolation associated with the COVID-19 pandemic have led to an increase in the number of patients with temporomandibular disorder (TMD) and bruxism. A total of 60.8% of them reported that their facial pain started in the past three months, while 51.4% of these individuals reported that their symptoms worsened in the past month and were associated with increased pain due to lockdown and experienced stress. The results of this study seem to support the hypothesis that the stress during the lockdown influenced the onset of TMD and facial pain [2]. Kim and co-authors showed that more patients reported pain from the palpation of the masticatory muscles during the pandemic, while this number decreased for the neck and temporomandibular joint muscles [27]. Studies by Di Giacomo et al. and Kim et al. also point to the adverse psychological effects of the COVID-19 pandemic on people with temporomandibular joint dysfunction (TMD) [28]. In our study, similar parameters were evaluated. Pain is very often the first symptom observed by patients and the main reason for seeking care and treatment. According to the IASP (International Association for the Study of Pain) classification, we distinguish different types of pain, which can overlap and intermingle. Nociceptive pain is the result of tissue damage and irritation of nociceptors; neuropathic pain arises as a result of the damage or disease of elements of the nervous system, while nociceptive pain is characterized by a disorder of pain sensation and exists despite the absence of damage to tissues and elements of the nervous system—a disruption of pain signal processing in the brain [29]. The effect of the co-occurrence of pain sensations of different nature is particularly evident in tension-type headaches and in pains of the masticatory organ, collectively categorised as the Craniofacial Pain of Musculoskeletal Origin group [29]. In our study, we observed a higher number of people reporting musculoskeletal pain in Group B than in Group A (especially among men), as well as an escalation of pain intensity in Group B, where women predominated in this aspect. Significant differences were noted in pain symptoms in the temporomandibular joint region between both Group A and B (*p* = 0.0002) and between genders in Group A and B. Both women and men experienced a heightened frequency of headaches in Group B. The incidence of otologic symptoms increased in Group B in the ear and/or preauricular area pain, which reveals clinical potential for this area.

Emodi-Perlman et al. studied the impact of the pandemic on the frequency and severity of orofacial pain by analysing maxillofacial conditions, such as temporomandibular disorders (TMD) and bruxism. The study was conducted with online surveys using anonymous questionnaires in two countries—Poland (n = 1092) and Israel (n = 700) [9]. The authors used the 3Q/TMD questionnaire to collect data. The results showed that the coronavirus pandemic has caused significant adverse effects on the psychoemotional status of both Israeli and Polish populations, resulting in the intensification of their bruxism and TMD symptoms. Although the study populations in Poland and Israel show cultural and demographic differences, the similarity of the study tools and time point allows the above work to be referenced [9]. For our study, 202 patients were enrolled, in whom, in addition to the Oral Behaviours Checklist (OBC) questionnaire, a specialised dental examination of the structures of the masticatory organ was performed each time using the DC/TMD questionnaire with the simultaneous use of the pain intensity scale from the RDC/TMD. The literature indicates that generalised anxiety disorder (GAD) is closely related to gender, with levels of stress and anxiety being higher in women [30]. According to Więckiewicz, women under 28 years of age, especially those who were unmarried, less educated, and living in Europe, were a group at higher risk of indirect health effects of the pandemic (development/exacerbation of bruxism and headaches) [31]. Our study showed that women in both Group A and B were more likely to report higher levels of pain severity in all muscles tested (especially the temporal muscle). However, an increase in the frequency of headaches was reported among men in Group B (Group A with 40.6% vs. Group B with 67.3%). The differences were observed in the intensity of pain in the temporomandibular joint area between women in Group A and B as well as between men in the study groups. The role of gender is important in the study because the majority of patients with TMD and headaches are women. Women are more susceptible to the effects of prolonged stressful situations, e.g., pandemic [9]. Our results, indicating an increase in the prevalence of headaches and pain in the temporomandibular joint area in men, would need to be further refined, e.g., the time they spent in front of the screen (on remote work), an assessment of their family’s financial situation and level of stress related to their sense of responsibility, and analysis of leisure activities (computer games). It is important to note the limited size of the study groups related to the pandemic situation. The co-occurrence of TMD and otological symptoms (such as tinnitus, ear pain, hypersensitivity to sounds, hearing loss, ear fullness, and dizziness) is often discussed in the literature [23,32,33,34].

There are several theories to explain the co-occurrence of otological symptoms in the context of TMD. The shared innervation of the maxilla, lower teeth, floor of the mouth, tongue, pharynx, larynx, and external ear via the trigeminal and vagus nerves explains the radiation of pain [35,36]. The close anatomical proximity of the ear and TMJ structures can also lead to the irritation of structures within the TMJ fossa, and chronic trauma to the tympanic cord (due to posterior displacement of the mandibular condyle) can cause contraction of the stapedius muscle and immobilisation of the stapes [35,36].

Any spatial changes within the TMJ, such as the backward displacement of the coronoid process (due to tooth loss in the support zones) can lead to injury to the anterior tympanic artery, resulting in disturbances in the nutrition of the tissues of the middle ear mucosa, skin, and outer layer of the tympanic membrane and observed hearing loss or tinnitus [35,36]. The prevalence of otological symptoms in patients with TMD and parafunctional occlusion varies widely (2–82.4%) [34,36]. In our study, the frequency of reported otologic symptoms increased in Group B, in both men and women. The most commonly reported otologic symptom in both groups was ear pain and/or preauricular pain. A comparison of the frequency of otological symptoms in groups A and B showed a non-significant increase, but potentially of clinical significance, in both women and men (*p* = 0.0058). Given the frequency of co-occurrence of masticatory dysfunction symptoms and otological symptoms, it seems reasonable to extend the dental history of symptoms of ear origin. However, it is important to exclude audiological/laryngological causes of the reported complaints, e.g., earwax as a cause of noise and fullness in the ear and inflammation of the external auditory canal as a cause of pain. The co-occurrence of bruxism and mandibular muscle tension with headaches, both primary (migraine and TTH) and secondary (acute and chronic post-traumatic headaches), was indicated [37,38]. According to the new diagnostic criteria of the International Classification of Headache Disorders, 3rd edition (ICHD-3), myofascial pain in the jaw region and headaches are correlated, and a bruxism headache is a separate headache type, coded 11.7 [39].

Assessment of the temporal relationship between headaches and bruxism is recommended to confirm that a headache is exacerbated during episodes of injurious occlusal parafunctions. The most common form of a headache is a tension-type headache (TTH)—prolonged, continuous, dull, aching pain of variable intensity occurring in the temporal, frontal, and suboccipital regions. The ethology of TTH emphasises the association between episodes of bruxism and headaches. Bruxism may also be an important contributor to the development of trigger points in the head and neck, which then leads to and/or contributes to the development of TTH and myofascial headaches (MHAs) [40]. Our study showed an increase in the severity of symptoms originating from the masticatory muscles, along with an increased incidence of pandemic headaches, in both men and women. However, in the study reported here, the frequency of headaches in males was significantly higher and was particularly related to the extraocular location. This is an interesting observation due to the well-known phenomenon of a higher prevalence of headaches and bruxism in women [41]. However, the changing nature of the work should not be overlooked. Working online via Internet platforms has become a new way of working during the COVID-19 pandemic, with a significant increase in the amount of time spent in front of a monitor screen in a sedentary position. There is something of a vicious circle between the increase in bruxism, muscle pain, and headaches during the pandemonium and the anxiety, stress, and changes in head and neck muscle tone caused by remote working—inappropriate posture and increased time spent in front of a screen associated with the shift to online working [42,43]. An unsuitable home office environment predisposes people to adopt inappropriate positions that can cause pain and musculoskeletal changes, particularly in the neck or cervical spine [44]. The results of the Prieto-González study showed that the cervical spine had the highest prevalence of musculoskeletal symptoms between December 2020 and January 2021 [42]. This nerve connection between the C1-C3 nerves and trigeminal nucleus is believed to be the cause of pain directed to the head. Aseptic inflammation and neurotransmission within the C-fibre, caused by cervical disc pathology, are believed to be responsible for the onset and exacerbation of pain in cervicogenic headaches [5]. It is noteworthy that there was a reduction in the age range in Group B by approximately 10 years compared to Group A (26.04~36.46 years). Group B showed a higher proportion of younger patients, particularly in the 18–23 age range. This could be attributed to the increased time spent studying online, while patients aged between 30 and 40 years were often working in hybrid mode or even fully on-site during the pandemic. Psychological aspects also played a key role, including the ability to cope with stress and mental resilience in times of danger, skills that are still developing in young people [45]. Carrillo-Diaz et al. point to a strong sense of isolation and a significant decrease in social activity among Spanish adolescents during the lockdown, which consequently led to increased insecurity and anxiety [46].

They observed an increase in symptoms of masticatory muscle pain in the age group. The author attributed this to an increased compulsive use of mobile devices, the internet, and social media during the COVID-19 pandemic. The aspect above would require an extended closer analysis to better understand the observed effect.

### Limitations of the Study

The present study, however, has several limitations due to the adopted study design. It is known that muscle tension changes depending on the emotional state (such as anxiety, anhedonia, and depression). Emotions play a significant role in TMD and are connected with the condition of the masticatory system and headaches. As a result, a potential limitation is that the study did not examine the patients’ exact emotional state or the impact of the severity of comorbid depression or anxiety. Any treatment by antidepressants (lengths of time and at different doses) in individuals was not evaluated, so the treatment factor was treated as a limitation. However, targeted psychological examinations would have involved a significant extension of the visit time; during the pandemic, the examination had to be as short as possible to provide the patient with only specific assistance.

Secondly, there is no information on the type of job performed by patients and the amount of hours lost spent in front of a computer screen while working on the Internet; also the time devoted to physical activity before and during the pandemic remains unknown. The above data may demonstrate a possible impact on the development or exacerbation of muscle tension due to changes in daily activity habits. This would allow for a more detailed diagnosis of head pain.

Moreover, lack of appropriate physical activity, prolonged time in an incorrect body position, and even disruption of the daily rhythm and changes in the organization of family life in the online work system may also contribute to the occurrence of headaches, generating a feeling of anxiety. It must be assumed that some patients were certainly already suffering from headaches before the lockdown, given the data on the epidemiology of headache types.

Finally, the study consisted of two independent groups of patients from before and during the pandemic. The study did not allow the evaluation of causation but only differences between groups because of the COVID-19 pandemic.

To confirm causation—the effect of the pandemic—other studies should trace the change in masticatory status and the characteristics of concomitant symptoms in an identical group of patients before and after the pandemic.

## 5. Conclusions

The conclusions are as follows:

1. The COVID-19 pandemic affected the incidence and severity of masticatory muscle pain and associated complaints.
2. A decrease in the age of patients reporting complaints of masticatory mm pain was observed during the COVID-19 pandemic.
3. An increase in the frequency of headaches was observed in the male group during the pandemic, while in women there was an increase in palpation tenderness of masticatory muscles.

## Figures and Tables

**Table 1:** Occurrence of pain in the masticatory muscles during palpation examination with division by sex (right and left side together)—no pain (intensity of pain = 0) vs. pain (intensity of pain = 1 + 2 + 3). Considered as significant at *p* < 0.0042 (Bonferroni correction).

	A		B		*p*
Intensity of Pain	0	1 + 2 + 3	0	1 + 2 + 3	
Mm. masseter					
all	50	152	18	184	<0.0001
F	6	122	4	136	0.5266
M	44	30	14	48	<0.0001
Mm. temporalis					
all	36	166	16	186	0.0044
F	8	120	4	136	0.2399
M	28	46	12	50	0.0369
Mm. medial pterygoid					
all	38	164	20	182	0.0153
F	10	118	2	138	0.0157
M	28	46	14	188	<0.0001
Mm. lateral pterygoid					
all	20	182	14	188	0.3705
F	6	122	4	136	0.5266
M	14	60	10	52	0.8219

**Table 2:** Intensity of masticatory muscle pain (0–3) during palpation examination with division by sex (right and left side together). Considered as significant at *p* < 0.0007 (Bonferroni correction).

	N	Left and Right Muscles of Group A				Left and Right Muscles of Group B				*p*	Comparison					
Muscle Examined	Pain Intensity	0	1	2	3	0	1	2	3		0 vs. 1	0 vs. 2	0 vs. 3	1 vs. 2	1 vs. 3	2 vs. 3
masseter	all	50	124	28	0	18	94	78	12	<0.0001	0.0169	<0.0001	<0.0001 *	<0.0001	0.0002	0.0667 *
	F	6	104	18	0	4	60	66	10	<0.0001	1 *	0.0239 *	0.0216 *	<0.0001	0.0002 *	0.2399 *
	M	44	20	10	0	14	36	10	2	<0.0001	<0.0001	0.0921	0.1356 *	0.3928	0.5402 *	0.5402 *
temporalis	all	36	126	40	0	16	68	102	16	<0.0001	0.5631	<0.0001	<0.0001	<0.0001	<0.0001	0.0150 *
	F	8	86	34	0	4	54	70	12	<0.0001	1 *	0.0333 *	0.002692 *	0.0001	0.0001	0.0261 *
	M	28	40	6	0	12	14	32	4	<0.0001	0.7949	<0.0001	<0.0001 *	<0.0001	0.0144 *	1 *
medial pterygoid	all	38	94	66	4	20	82	86	14	0.002	0.1068	0.0126	0.0075	0.1008	0.0234	0.1008
	F	10	58	56	4	2	70	70	10	0.05	0.0386	0.0386	0.0386	0.7509	0.3559	0.3559
	M	28	36	10	0	8	22	16	4	<0.0001	<0.0001	<0.0001	<0.0001	0.0007	0.0043 *	0.3589 *
lateral pterygoid	all	20	102	70	10	14	84	86	18	0.0820	0.6673	0.2098	0.1399	0.1399	0.1400	0.4417
	F	6	72	50	8	4	60	64	14	0.1	0.7545 *	0.3992 *	0.2783 *	0.2513	0.2513	0.3992
	M	14	30	20	2	10	24	22	4	0.8690	1	1	1 *	1	1 *	1 *

*p* value Chi^2^/* Fisher (Cochran unfulfilled condition).

**Table 3:** The severity of pain in the temporomandibular joint area in groups A and B, by gender.

Intensity of Pain in the TMJ Region	0	1	2	3
TMJ group A				
F	92 71.88%	32 25.00%	4 3.13%	0 0.00%
M	57 77.03%	14 18.92%	3 4.05%	0 0.00%
TMJ group B				
F	80 57.14%	46 32.86%	12 8.57%	2 1.43%
M	35 56.45%	19 30.65%	8 12.90%	0 0.00%

**Table 4:** Acoustic symptoms in the examined groups by sex. Both TMJ (right and left) were considered. Considered as significant at *p* < 0.025 (Bonferroni correction).

Acoustic Symptoms	TMJ Group A		TMJ Group B	
	F	M	F	M
Yes	33 (25.78%)	22 (29.73%)	47 (33.57%)	19 (30.65%)
No	95 (74.22%)	52 (70.27%)	93 (66.43%)	43 (69.35%)
*p*	*p* = 0.54357		*p* = 0.68255	

**Table 5:** Comparison of the occurrence of acoustic symptoms by sex. Both TMJ (right and left) were considered. Considered as significant at *p* < 0.025 (Bonferroni correction).

	Acoustic Symptoms				
	Yes	No		Yes	No
Group A F	33 25.78%	95 74.22%	Group A M	22 29.73%	52 70.27%
Group B F	47 33.57%	93 66.43%	Group B M	19 30.65%	43 69.35%
*p* = 0.16390			*p* = 0.90776		

**Table 6:** Comparison of the frequency of otological symptoms in Group A and B, considering sex and type of symptoms. Considered as significant at *p* < 0.0042 (Bonferroni correction).

Studied Group n	Overall Otological Symptoms n (%)	Isolated Tinnitus n (%)	Feeling of Fullness in the Ear n (%)	Ear and/or Preauricular Area Pain n (%)
Whole group A *n = 101*	31 (30.69%)	12 (11.88%)	8 (7.92%)	14 (13.86%)
F *n* = 64	21 (32.81%)	7 (10.93%)	7 (10.93%)	7 (10.93%)
M *n* = 37	10 (27.03%)	5 (13.51%)	1 (2.70%)	7 (18.91%)
Whole group B *n = 101*	44 (43.56%)	19 (18.81%)	9 (8.91%)	24 (23.76%)
F *n* = 70	30 (42.86%)	11 (15.71%)	5 (7.14%)	13 (18.57%)
M *n* = 31	14 (45.16%)	8 (25.81%)	4 (12.90%)	11 (35.49%)
Difference (%), *p* value				
Whole group A vs. whole group B	12.87% *p* = 0.058	6.93% *p* = 0.175	0.99% *p* = 0.798	9.90% *p* = 0.073
F–A vs. F–B	10.05% *p* = 0.231	4.78% *p*-value = 0.417	3.79% *p*-value = 0.442	7.64% *p* = 0.213
M–A vs. M–B	18.13% *p* = 0.121	12.30% *p* = 0.202	10.20% *p* = 0.111	16.58% *p* = 0.125

**Table 7:** Headache intensity in Group A and B, divided by sex.

Pain Intensity	0	1	2	3
Whole group A	60 59.41%	20 19.81%	19 18.81%	2 1.98%
F	28 43.75%	18 28.12%	16 25.00%	2 3.12%
M	32 86.49%	3 8.11%	2 5.40%	0
Whole group B	33 32.67%	33 32.67%	31 30.69%	4 3.96%
F	20 28.57%	25 35.71%	22 31.43%	3 4.28%
M	13 41.93%	10 32.26%	7 22.58%	1 3.22%

**Table 8:** Comparison of headache prevalence in groups A and B with predominant localization by gender. Considered as significant at *p* < 0.0056 (Bonferroni correction). Statistically significant results are marked in bold.

Studied Group n	Headache n (%)	Temporal Headache n (%)	Other Location (Occipital, Parietal, Frontal, or Banded) n (%)
Whole group A	41 (40.59%)	29 (28.71%)	12 (11.88%)
F	36 (56.25%)	25 (39.06%)	11 (17.19%)
M	5 (13.51%)	4 (10.81%)	1 (2.71%)
Whole group B	68 (67.33%)	40 (39.60%)	28 (27.72%)
F	50 (71.43%)	32 (45.71%)	16 (22.86%)
M	18 (58.06%)	8 (25.81%)	12 (38.71%)
Difference (%), *p* value			
Group A vs. Group B	26.74% *p* < **0.001**	10.89% *p* = 0.103	15.84% *p* = **0.005**
F–A vs. F–B	15.18% *p* = 0.070	6.65% *p* = 0.442	5.67% *p* = 0.413
M–A vs. M–B	44.55% *p* < **0.001**	15.00% *p* = **0.027**	36.00% *p* < **0.001**

## Data Availability

Data supporting the findings of this study are available within the article.
